# Standard Tone Stability as a Manipulation of Precision in the Oddball Paradigm: Modulation of Prediction Error Responses to Fixed-Probability Deviants

**DOI:** 10.3389/fnhum.2021.734200

**Published:** 2021-09-28

**Authors:** Iria SanMiguel, Jordi Costa-Faidella, Zulay R. Lugo, Elisabet Vilella, Carles Escera

**Affiliations:** ^1^Brainlab-Cognitive Neuroscience Research Group, Department of Clinical Psychology and Psychobiology, University of Barcelona, Barcelona, Spain; ^2^Institute of Neurosciences, University of Barcelona, Barcelona, Spain; ^3^Institut de Recerca Sant Joan de Déu, Esplugues de Llobregat, Spain; ^4^Hospital Universitari Institut Pere Mata, Universitat Rovira i Virgili (URV), Institut d’Investigació Sanitària Pere Virgili (IISPV), Reus, Spain; ^5^Centro de Investigación Biomédica en Red en Salud Mental (CIBERSAM), Madrid, Spain

**Keywords:** uncertainty, precision, prediction error, mismatch negativity (MMN), deviance detection, predictability, oddball

## Abstract

Electrophysiological sensory deviance detection signals, such as the mismatch negativity (MMN), have been interpreted from the predictive coding framework as manifestations of prediction error (PE). From a frequentist perspective of the classic oddball paradigm, deviant stimuli are unexpected because of their low probability. However, the amount of PE elicited by a stimulus can be dissociated from its probability of occurrence: when the observer cannot make confident predictions, any event holds little surprise value, no matter how improbable. Here we tested the hypothesis that the magnitude of the neural response elicited to an improbable sound (D) would scale with the precision of the prediction derived from the repetition of another sound (S), by manipulating repetition stability. We recorded the Electroencephalogram (EEG) from 20 participants while passively listening to 4 types of isochronous pure tone sequences differing in the probability of the S tone (880 Hz) while holding constant the probability of the D tone [1,046 Hz; p(D) = 1/11]: Oddball [p(S) = 10/11]; High confidence (7/11); Low confidence (4/11); and Random (1/11). Tones of 9 different frequencies were equiprobably presented as fillers [p(S) + p(D) + p(F) = 1]. Using a mass-univariate non-parametric, cluster-based correlation analysis controlling for multiple comparisons, we found that the amplitude of the deviant-elicited ERP became more negative with increasing S probability, in a time-electrode window consistent with the MMN (ca. 120–200 ms; frontal), suggesting that the strength of a PE elicited to an improbable event indeed increases with the precision of the predictive model.

## Introduction

According to current models that view the brain as a Bayesian inference system, our experience of the world stems from internal representations of the statistical regularities of the sensory input. These internal representations embody our experience and prior knowledge about the world, and the associated expectations. Based on these representations, internal forward models continuously make predictions regarding the sensory input (Friston, 2010). Predictions are compared with incoming sensory information and prediction error (PE) is used to adjust the internal representations. This comparison process and the ensuing generation of PE signals is also referred to as sensory deviance detection, and it is reflected in electrophysiological responses, most notably in the mismatch negativity (MMN; Garrido et al., 2009). Today, deviance detection is widely accepted as a general principle of brain function (Friston, 2010; Escera and Malmierca, 2014; Malmierca et al., 2014).

An aspect of this process which is much less well-established in the deviance detection literature is the proposal that it is flexibly adjusted depending on the estimated precision of the sensory signals, or in other words, the confidence that can be placed in the current internal models and the predictions derived from them. Specifically, it is proposed that the gain of the PE signals is modulated (“precision-weighted”) by their expected precision, thereby adjusting the impact that the PE has in terms of updating the internal representations (Feldman and Friston, 2010; Schröger et al., 2015). This is critical for the proper formation and updating of predictive models under different contexts and levels of noise, avoiding issues like overfitting, and allowing a dynamic adjustment of the balance between the weight placed on priors and the weight placed on sensory evidence when interpreting sensory input. Precision-weighting of the gain of the PE signal is also proposed to be the mechanism through which attention operates to modulate sensory responses (Feldman and Friston, 2010). Moreover, it has been proposed that dysfunctional precision-weighting might be a critical factor in schizophrenia and autism, in which the balance between the weight placed on priors and evidence would be skewed toward the priors in the former and toward the evidence in the latter (Adams et al., 2013; Lawson et al., 2014, 2017). Thus, the concept of precision or confidence appears to be a central aspect of generating and using internal models, crucial in determining our experience through its influence on perception and attention.

Nevertheless, the concept of confidence or precision is somewhat elusive and has been rarely operationalized in a clear way in deviance detection studies. So far, it seems unclear how to measure confidence and investigate it with a simple paradigm that is also applicable to clinical settings. The aim of this report is to propose a simple manipulation that taps into precision or confidence based on the most common design to investigate deviance detection, the oddball paradigm, allowing us to investigate the hypothesis that sensory deviance detection signals are precision weighted.

In the typical oddball paradigm used to study deviance detection, two stimuli are presented with differing probabilities; an infrequent “deviant” stimulus is interspersed among the repeating presentation of the frequent “standard” stimulus. When the electrophysiological responses elicited by the deviant are compared to those elicited by the standard, a negative deflection can be observed on the difference waveform in the 100-200 ms latency range: the mismatch negativity (MMN; Näätänen, 1992). Thus, the MMN signals the detection of a change in the sensory stream. Since the discovery of the MMN, thousands of studies have used variations of the oddball paradigm, applying the MMN to study a wide range of issues in basic and clinical research, proving to be a powerful tool to study brain function (Näätänen et al., 2007, 2011, 2012). For the MMN to continue to be so useful, our understanding of the underlying MMN-generating process and significance must continue to be updated and evolve (Winkler, 2007).

Indeed, there has been a substantial progression on the explanatory theories regarding the type of computation indexed by the MMN-generating process. Initially, the sensory memory trace hypothesis proposed that each incoming stimulus is compared with the trace of the preceding stimuli stored in sensory memory and that MMN is elicited when the incoming stimulus differs (Näätänen, 1992). An alternative explanation proposed that the MMN is the result of the differential state of refractoriness or adaptation of the neural populations responding to the standard and deviant stimulus, with the standard population being more refractory due to the high rate of responses to the repeating standard, and thus eliciting a diminished response (“release from refractoriness,” Näätänen, 1990, 1992), or “N1 adaptation hypothesis” (Jääskeläinen et al., 2004; May and Tiitinen, 2010) compared to the deviant. Currently, it is generally acknowledged that refractoriness differences underlie part of the effects measured in most of the classic deviance detection studies unless this aspect is properly controlled (Näätänen et al., 2005; Escera and Malmierca, 2014). Nevertheless, the predominant view is that there exists a unique deviance detection process indexed beyond refractoriness differences (the “true” MMN). Building up on this idea, the sensory memory trace hypothesis evolved into considering the trace against which each incoming stimulus is compared more of an abstract representation of a regularity, rather than a literal trace of the standard stimulus. The idea of regularity representations facilitated a transition from memory-based to prediction-based explanations, proposing that the comparison is not to a memory trace, but rather to a prediction generated on the basis of the regularity. Perhaps the currently best accepted view of the MMN is the model adjustment hypothesis, which also highlights the predictive model, proposing that the MMN-generating process has a direct role in the building of the predictive internal representation itself, rather than simply signaling deviance detection (Winkler and Czigler, 1998; Winkler, 2007). In this view, the MMN reflects the updating of the internal representation on the basis of how well the incoming stimuli match the predictions generated by the predictive model. However, while these last models stress the predictive aspect, they did not define how specifically the predictive representation is formed and applied. More recently, MMN has been interpreted from the predictive coding perspective as a manifestation of PE (Garrido et al., 2009; Wacongne et al., 2011; Lieder et al., 2013; Schröger et al., 2015; Stefanics et al., 2018), placing the MMN-generating process within a wider conceptualization of the brain as a Bayesian inference system (Knill and Pouget, 2004; Friston, 2010) and thus providing a detailed explanation of the computations involved in the underlying inference process.

The different MMN models emphasize different aspects when it comes to understanding exactly what the *deviance* in deviance detection is, and thus outlining the factors that might influence MMN elicitation and amplitude. From a rather simple frequentist perspective of the classic oddball paradigm, the deviance associated with an event relates to its improbability, given a prediction of the occurrence of all possible events. Thus, in this view, the differential processing of deviant stimuli is determined exclusively by their low probability. This interpretation fits well with the N1-adaptation hypothesis, in which the effects would be due solely to differential base rate probability of the standard and deviant. However, from a Bayesian perspective PE (and thus MMN) reflects a violation of expectations, and can be related in a straightforward manner to the concept of Bayesian surprise (Ostwald et al., 2012). Bayesian surprise quantifies how incoming data affects an observer, by measuring the difference between the observer’s beliefs before and after receiving the new data. New data that is difficult to integrate into the current explanatory model (i.e., the observer’s beliefs) requires that significant changes are made to the model, thus yielding a high value of Bayesian surprise (Itti and Baldi, 2009). This perspective dissociates the amount of PE (surprise) elicited by a stimulus from its probability of occurrence, and also fits well with the model adjustment hypothesis of MMN (Winkler, 2007). The Bayesian perspective on surprise also stresses the importance of the observer’s beliefs: when the observer cannot make confident predictions, any event holds little surprise value, no matter how improbable it is by itself.

In predictive coding models of brain function, confidence in the predictions derived from the internal model is tied to the concept of precision. Predictive coding proposes that the prediction error signal is weighted by an estimate of its expected precision, which inversely relates to the prediction error’s variability (Feldman and Friston, 2010). This precision-weighting mechanism allows adjusting the relative weights of prior beliefs and sensory evidence in the inference process considering contextual factors, such as the amount of noise. Thus, the magnitude of sensory deviance detection signals elicited by a highly improbable deviant stimulus should reflect the confidence (precision), such that it should be down-weighted when contextual factors lead to highly variable signals. In other words, a highly improbable event will elicit less surprise when the situation does not allow constructing an internal model that reliably predicts the stimulation.

In sum, modern perspectives on the MMN-generating process place the concept of confidence or precision as a central parameter in the elicitation of MMN. However, until quite recently, among the myriad of studies on MMN there have been surprisingly very few that directly addressed this aspect. Nevertheless, classic MMN literature has shown that the MMN is modulated by factors that reflect the clarity or the certainty of a change (Fitzgerald and Todd, 2020). First, MMN will only be elicited to deviants presented with a probability of 0.30 or below (Kujala et al., 2007), and MMN amplitude increases with decreasing probabilities of the deviant (Näätänen, 1990, 1992). It is well-established that the MMN is larger for deviants that are more physically different (Winkler et al., 1992; Tiitinen et al., 1994; Amenedo and Escera, 2000; Daikhin and Ahissar, 2012), differ in more dimensions (Schröger and Wolff, 1998) or are more discriminable (Sams et al., 1985) from the standard (Näätänen, 1990, 1992). Much less research has focused on exploring the impact of the way the regularity is presented, that is, the characteristics of the standards rather than the deviants. Nevertheless, there is evidence indicating that the stability or strength of the regularity, or the amount of evidence gathered to support it, affects MMN amplitude. MMN increases after a greater number of repetitions of the standard (Baldeweg et al., 2004; Costa-Faidella et al., 2011a,b), after a longer period of stable regularity (Todd et al., 2011) or when the rate of standard repetitions is higher (with shorter ISIs; Pekkonen et al., 1995). Importantly, not only the amount of evidence collected for the regularity but also the clarity of this evidence plays a role. In this sense, factors that diminish the information extracted from the standard attenuate MMN (e.g., backward masking, Winkler and Näätänen, 1992), and introducing some variability in the specific characteristics of the repeating standard stimulus also decreases the amplitude of the MMN (Winkler et al., 1990).

All in all, although there is evidence indicating that confidence or precision may play an important role in the MMN-generating process, a simple dedicated paradigm is lacking that would allow to measure the effects of precision understanding the MMN as an index of a Bayesian inference process. Such a paradigm should allow isolating confidence without being confounded by refractoriness, which is tied to the deviant probability. Moreover, a clear operational definition of confidence applied to the oddball paradigm is missing to facilitate research in this aspect and hopefully lead to a better understanding of the MMN-generating process.

To investigate the influence of precision in sensory deviance detection signals, we propose a new oddball paradigm in which we vary the confidence on the model (inferred from the regularity established by the repetition of the standard stimulus), by manipulating the stability of the standard stimulus, while holding the deviant probability constant. To isolate effects of precision not confounded by refractoriness differences, we focus on the analysis of the deviant stimuli, which should elicit a precision-weighted PE signal reflecting the deviance detection process. If the MMN reflects the probability of the deviant stimulus, responses to the deviant should not differ between conditions. On the contrary, if it is a prediction error signal weighted by the confidence given by the overall variability of the stimulation, we would expect the amplitude of the deviant responses to be graded by the probability of the standard tone.

## Materials and Methods

### Participants

Twenty-five healthy volunteers with no self-reported history of neurological, psychiatric, or hearing impairment and with normal or corrected-to-normal visual acuity participated in the experiment. From this sample, five participants had to be excluded due to problems during the recording session (*N* = 2) or large artifacts in the Electroencephalogram (EEG) signal (*N* = 3), resulting in a final sample of 20 participants included in the study (mean age: 34.5 years; age range: 21–55 years; 8 males; all right-handed). All volunteers gave written informed consent in accordance with the guidelines of the Clinical Research Commission of the Hospital Universitari Institut Pere Mata and the Ethics Committee of the Institut d’Investigació Sanitària Pere Virgili before their participation and after the procedures were explained to them. The study conformed to the Code of Ethics of the World Medical Association (Declaration of Helsinki) and was approved by the Clinical Research Commission of the Hospital Universitari Institut Pere Mata, the Drug Research Ethics Committee of the Institut d’Investigació Sanitària Pere Virgili and the Bioethics Committee of the University of Barcelona. Recordings were performed at the Hospital Universitari Institut Pere Mata.

### Auditory Stimuli

Eleven pure tones (44.1 kHz sampling rate; 50 ms duration; 5 ms hanning windowed rise/fall ramps) of different frequencies corresponding to musical notes, from A4 as the lowest pitch and Eb7 as the highest, spaced in steps of 3 semitones (440; 523.25; 622.25; 739.99; 880; 1046.5; 1244.51; 1479.98; 1760; 2093; and 2489.02 Hz), were generated with Matlab (R2020a; Mathworks) and delivered binaurally via Sony MDR-ZX110 headphones at 70 dB SPL using Psychtoolbox-3 functions implemented in Matlab environment [Psychophysics Toolbox Version 3 (PTB-3)] (Brainard, 1997; Pelli, 1997; Kleiner et al., 2007).

### Sound Sequences

**FIGURE 1:**
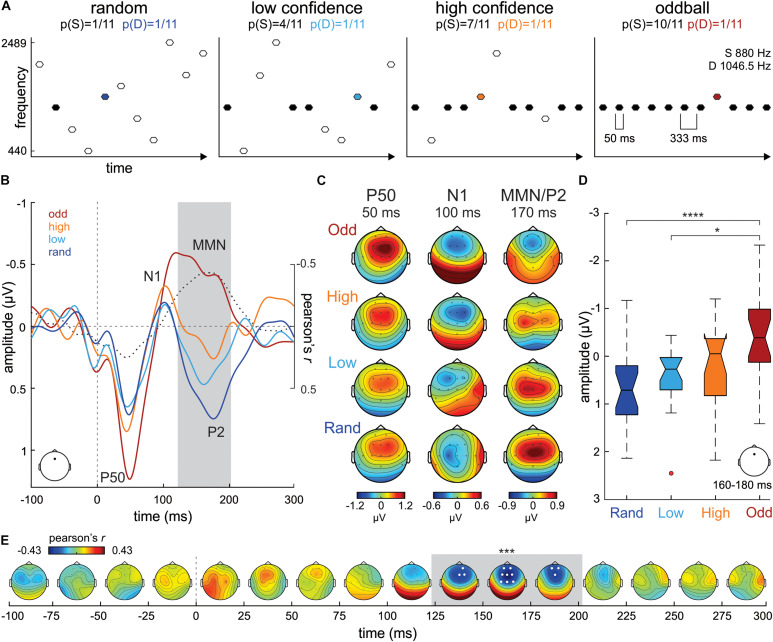
**(A)** Experimental design. **(B)** ERP waveforms from Fz electrode evoked to D stimuli in the Oddball (odd; *dark red*), High confidence (high; *orange*), Low confidence (low; *cyan*) and Random (rand; *dark blue*) conditions. Correlation values (Pearson’s *r*) between S tone probability and D tone ERP amplitudes at each time point are plotted in a dotted black line. The gray shaded area marks the temporal extent of the significant cluster of correlation values (122–202 ms). **(C)** P50, N1 and MMN/P2 scalp potential distribution maps per condition separately. **(D)** Boxplot series illustrating the distribution of mean amplitude ERP values extracted from Fz around the maximum correlation peak (170 ms) in our sample of participants, separately per condition. The boxplots represent the median value (middle line), the interquartile range (full box) and the extreme values (whiskers; outliers are plotted as separate dots). Significance of *post hoc* tests: ****p < 0.001; *p < 0.05. **(E)** Time-electrode evolution of Pearson’s *r*-values. The gray shaded area marks the temporal extent (122–202 ms) of the significant (***p < 0.005) cluster of correlation values, while the white dots (electrodes) denote its spatial extent.

Auditory stimuli were arranged in four separate sequences (see Figure 1A), each containing 1650 pure tones delivered randomly at 333 ms SOA. Each sequence constituted one experimental block (i.e., one condition; ca. 9 min duration). Sequences differed mainly in the probability of appearance of the 880 Hz tone [from now on termed Standard (S)]: Oddball, p(S) = 10/11; High confidence, p(S) = 7/11; Low confidence, p(S) = 4/11 and Random (no tone repetition allowed), p(S) = 1/11. The probability of appearance of the 1046.5 Hz tone [from now on termed Deviant (D)] was kept at p(D) = 1/11 in all sequences. The remaining nine tones were presented as equiprobable fillers (from 440 to 2489.02 Hz, spaced in 3 semitone steps, excluding the D and S tones) with a combined probability of appearance of p(fillers) = 1−[p(S) + p(D)]. Each sequence was created by concatenating 150 microsequences of 11 tones (150^∗^11 = 1650), generated according to the required characteristics. If a microsequence started with the same tone as that appearing at the previous microsequence ending (except for the S tone in the Oddball, High confidence and Low confidence sequences), a different microsequence was generated to avoid repetition. Albeit acknowledging that the S/D terms may not be appropriate for a sequence such as the Random one, in which all 11 tones are presented equiprobably, we decided to follow the traditional terminology of human auditory ERP studies on deviance detection (Näätänen, 1992; Näätänen et al., 2007) for consistency with past literature, readability, and because it reflects best our experimental manipulation of interest: the parametric variation of S probability.

### Procedure

During the EEG recording session, participants sat in a comfortable chair in a sound-attenuated room and listened passively to the four sound sequences, delivered in random order, while reading a book (or a magazine or newspaper) of their own choosing. The total duration of the recording session was 40 min approximately (4 blocks × 9 min + pauses), plus EEG recording preparation (around 20 min).

### EEG Recording and Preprocessing

EEG was continuously recorded from 16 Ag/AgCl electrodes and digitized at a sampling rate of 500 Hz by a BrainVision V-AMP amplifier (Brain Products, Germany) using the BrainVision Recorder version 1.21.0303 (Brain Products, Germany) acquisition software. Eleven electrodes were mounted in a nylon cap (EasyCap, Germany) at standard locations according to the international 10-20 system (Fp1, Fp2, F3, Fz, F4, T3, C3, Cz, C4, T4, Pz); additionally, two electrodes were positioned over the left and the right mastoids (M1 and M2, respectively), and three electrodes were used to record the electrooculogram [one placed below the left eye (VEOG); the remaining two placed at the outer canthi of the eyes (HEOG)]. The ground electrode was placed at AFz and the common reference electrode at the tip of the nose. All impedances were kept below 5 kΩ during the whole recording session.

Data preprocessing was performed offline using EEGlab v2021.0 software (Delorme and Makeig, 2004) running on Matlab R2020a. Data were bandpass filtered between 1 and 40 Hz (Kaiser window; β = 5.65; transition bandwidth = 0.5 Hz). Periods contaminated by non-stereotyped muscle artifacts were rejected by visual inspection. Independent component analysis decomposition was applied using the SOBI algorithm (Belouchrani and Cichocki, 2000). Independent components related to blinks, horizontal eye movements and heart rate, identified on the basis of their scalp topography and time course (Jung et al., 2000), were removed. After eliminating VEOG and HEOG channels from the set, artifact corrected data were cut in epochs from −0.1 to 0.3 s, time-locked to each auditory stimulus onset, and baseline corrected from −0.1 to 0 s. Epochs containing improbable data 3 SD above or below the mean probability distribution of values across all epochs were excluded (EEGlab’s function *pop_jointprob.m*). Epochs corresponding to the D tone and the closest preceding S tone were selected for further analyses. Across participants, the mean (and SD) of the number of included trials per condition was: Oddball, D tone, 134.4 trials (7.2), S tone, 135.3 trials (5.7); High confidence, D tone, 135.9 trials (5.6), S tone, 136.3 trials (5.2); Low confidence, D tone, 133.95 trials (9.7), S tone, 133.4 trials (6.5); Random, D tone, 135.45 trials (4.7), S tone, 135.4 trials (5.7). No significant differences were found between the number of trials used in each condition (D tone: Kruskal-Wallis test, χ^2^ = 0.5, *p* > 0.5, df = 3; S tone: Kruskal-Wallis test, χ^2^ = 2.35, *p* > 0.1, df = 3). Data was then converted to fieldtrip format (Oostenveld et al., 2011), epochs were averaged separately per participant, tone type and condition and the resulting ERPs were lowpass filtered at 25 Hz with a zero-phase forward and reverse 6th order Butterworth IIR filter (hamming window). Difference waves (DW) were computed by subtracting, per participant and condition, the S tone ERP from the D tone ERP.

### EEG Analyses

To investigate the influence of precision on deviance detection signals, we focused on the analysis of the D stimulus under different levels of precision, with the hypothesis that ERP amplitudes to the D tone would be modulated by the probability of the S tone. We computed a correlation analysis (Pearson’s correlation) introducing the probability of the S tone as the independent variable (i.e., predictor; 10/11, 7/11, 4/11, 1/11 corresponding to the *Oddball*, *High confidence*, *Low confidence* and *Random* conditions, respectively) and the ERP amplitudes to the D tone (in the 4 experimental conditions) as the dependent variable. In order to overcome the problem of multiple comparisons over electrodes (*n* = 13) and time points (from −0.1 to 0.3 s; 200-time points at 500 Hz sampling rate), a mass-univariate, two-dimensional (time, electrode) cluster-based correlation analysis was conducted, performed using a non-parametric randomization procedure (Maris and Oostenveld, 2007; in Fieldtrip, *ft_timelockstatistics* function with the options *cfg.statistic* = *“correlationT”* and *cfg.type* = *“Pearson”*). Neighboring electrodes were defined by the distance separating each other in a 2D projection of the montage, centering a 2.5 cm radius circle at each electrode and selecting those electrodes falling within. A minimum of two nearby electrodes was set per cluster. Correlation coefficient T-statistics were then computed at each time point and electrode (two-tailed) with the non-parametric Monte Carlo method. The Monte Carlo significance probability (*p*-value) was determined by calculating the proportion of 2D samples from 20,000 random partitions of the data that resulted in a larger test statistic than those on the observed test statistic. Then, clusters were created by grouping adjacent 2D points exceeding a significance level set to 0.05. The weighted cluster mass (Hayasaka and Nichols, 2004) was taken as the cluster-level statistic. The significance probability of the clusters was assessed with the described non-parametric Monte Carlo method. Values of *p* < 0.05, corrected for two-tailed tests, were considered significant. For each significant cluster we report its temporal spread, cluster statistic and *p*-value. To facilitate comparability of our results to previous MMN deviance detection studies, we complemented the analyses performed on D stimuli, analyzing the modulation caused by the probability of the S tone on the S tone ERP itself and on the D-S DW, following exactly the same statistical approach. However, note that differences in the S responses between conditions do not only reflect differences in precision, but also differences in refractoriness or adaptation as the manipulation of precision entails the manipulation of the S stimulus repetition rate. Therefore, we base our conclusions on the analysis of the D stimuli, whose probability was held constant across conditions.

## Results

Grand-average (*N* = 20) ERP waveforms evoked to the D tone (1046.5 Hz; probability of appearance in the sequence = 1/11) in the *Random*, *Low confidence*, *High confidence* and *Oddball* conditions, extracted from a frontocentral electrode (Fz), are illustrated in Figure 1B. As expected, the tone evoked prototypical P50 (ca. 50 ms) and N1 (ca. 100 ms) auditory ERP components in all conditions. A gradient in the ERP amplitude, becoming more negative with increasing S tone (880 Hz) probability across conditions (1/11, 4/11, 7/11 and 10/11 for the *Random*, *Low confidence*, *High confidence* and *Oddball* conditions, respectively), can be appreciated between ca. 120 and 200 ms, a time range consistent with that of MMN/P2 auditory ERPs. The scalp potential distribution maps of these ERP components are plotted in Figure 1C for each condition separately.

A mass-univariate correlation analysis between the probability of the S in the different experimental conditions (independent variable) and the amplitude of the ERP to the D tone (dependent variable), corrected for multiple comparisons in time and space (i.e., number of electrodes) using a cluster-based approach, yielded a significant fronto-central negative cluster between 122 ms and 202 ms (*wcm* = −247.48; *p* < 0.005; see Figure 1E), peaking at Fz electrode at 170 ms (*Pearson’s r* = −0.43; see Figure 1B), corroborating the observation that D tone ERP amplitudes become more negative as S probability increases. This result was supported by a further confirmatory non-parametric statistical analysis on the mean ERP amplitudes at Fz extracted from each subject in a 160 to 180 ms time window (20 ms around the correlation peak; Kruskal-Wallis test, χ^2^ = 16.59, *p* < 0.001, df = 3; see Figure 1D). *Post hoc* tests corrected for multiple comparisons (Tuckey-Kramer) revealed that D ERP amplitudes at Fz during that time range were significantly more negative in the *Oddball* condition than in the *Random* (*p* < 0.001) and in the *Low confidence* (*p* < 0.05) conditions.

**FIGURE 2:**
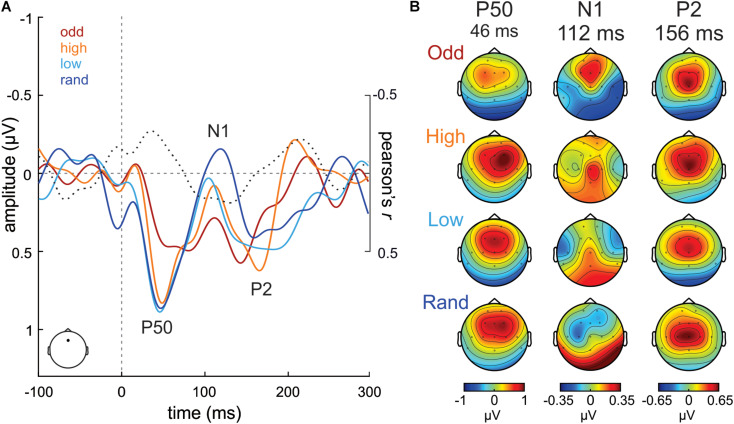
**(A)** ERP waveforms from Fz electrode evoked to S stimuli in the Oddball (odd; dark red), High confidence (high; orange), Low confidence (low; cyan) and Random (rand; dark blue) conditions. Correlation values (Pearson’s r) between S tone probability and S tone ERP amplitudes at each time point are plotted in a dotted black line. **(B)** P50, N1, and P2 scalp potential distribution maps per condition separately.

In order to evaluate the modulation that increasing a tone probability has on the activity evoked to that tone itself, the activity evoked to the S tones was submitted to the very same analysis. Grand-average ERP waveforms evoked to the S tone (880 Hz) in the Random, Low confidence, High confidence and Oddball conditions, extracted from Fz, are illustrated in Figure 2A. A gradient in the N1 amplitude (ca. 110 ms) can be observed, becoming less negative with increasing probability, as well as a reduced P50 (ca. 45 ms) in the Oddball condition as compared to the rest. The scalp potential distribution maps of these ERP components are plotted in Figure 2B for each condition separately.

However, these observations were not supported by statistical analyses, as the mass-univariate correlation analysis performed between the probability of the S in the different experimental conditions (independent variable) and the amplitude of the ERP to the S tone (dependent variable), corrected for multiple comparisons in time and space (i.e., number of electrodes) using a cluster-based approach, yielded no significant clusters.

**FIGURE 3:**
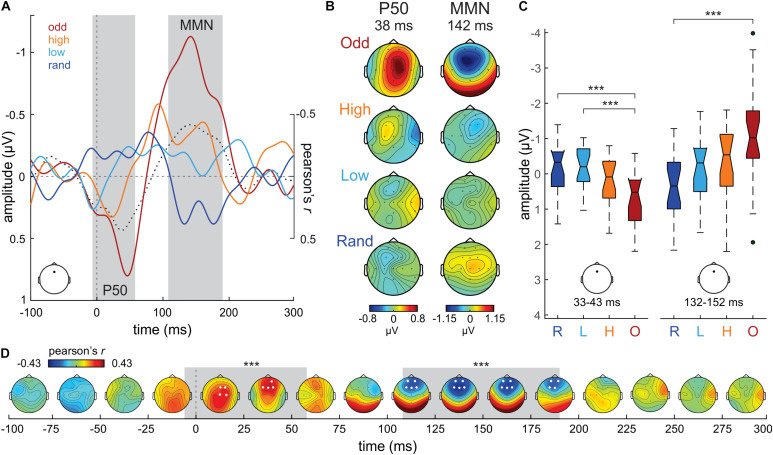
**(A)** Difference waveforms (D ERP—S ERP) from Fz electrode in the Oddball (odd; dark red), High confidence (high; orange), Low confidence (low; cyan) and Random (rand; dark blue) conditions. Correlation values (Pearson’s r) between S tone probability and DW ERP amplitudes at each time point are plotted in a dotted black line. The gray shaded areas mark the temporal extent of the significant clusters of correlation values (–6–58 ms; 108 to 190 ms). **(B)** P50 and MMN time range scalp potential distribution maps per condition separately. **(C)** Boxplot series illustrating the distribution of DW mean amplitude values extracted from Fz around the maximum correlation peaks (38 ms; 142 ms) in our sample of participants, separately per condition and P50/MMN time ranges. The boxplots represent the median value (middle line), the interquartile range (full box) and the extreme values (whiskers; outliers are plotted as separate dots). Significance of *post hoc* tests: ***p < 0.005. **(D)** Time-electrode evolution of Pearson’s *r*-values. The gray shaded areas mark the temporal extent (–6–58 ms; 108–190 ms) of the significant (***p ≤ 0.005) clusters of correlation values, while the white dots (electrodes) denote their spatial extent.

For completeness, we submitted the DW ERPs (D ERP – S ERP) to the same analysis. Grand-average DW ERPs in the Random, Low confidence, High confidence and Oddball conditions, extracted from Fz, are illustrated in Figure 3A. As expected from the activity patterns elicited to the D and S tones, the DWs exhibit an increase in positivity around the P50 time range (ca. 40 ms) with increasing S tone probability, as well as a prominent MMN (ca. 140 ms) in the Oddball condition. The negativity at the MMN time range gradually increases with increasing S tone probability. The scalp potential distribution maps of both DW peaks are plotted in Figure 3B for each condition separately.

The mass-univariate correlation analysis between the probability of the S in the different experimental conditions (independent variable) and the amplitude of the DW ERP (dependent variable), corrected for multiple comparisons in time and space (i.e., number of electrodes) using a cluster-based approach, yielded a significant central-frontocentral positive cluster between −6 ms and 58 ms (wcm = 166.71; *p* < 0.01; see Figure 3D), peaking at Fz electrode at 38 ms (Pearson’s *r* = 0.43; see Figure 3A), and a significant frontocentral negative cluster between 108 ms and 190 ms (wcm = −234.54; *p* < 0.005; see Figure 3D), peaking at Fz electrode at 142 ms (Pearson’s *r* = −0.42; see Figure 3A). These results corroborate the observations that DWs increase in positivity at the P50 time range and increase in negativity at the MMN time range with increasing S tone probability. Further confirmatory non-parametric statistical analyses on the mean DW amplitudes at Fz extracted from each subject confirmed these findings: P50 time range, 33–43 ms time window (10 ms around the correlation positive peak), Kruskal-Wallis test, χ^2^ = 15.49, *p* < 0.005, df = 3; see Figure 3C; MMN time range, 132–152 ms time window (20 ms around the correlation negative peak), Kruskal-Wallis test, χ^2^ = 14.26, *p* < 0.005, df = 3; see Figure 3C. *Post hoc* tests corrected for multiple comparisons (Tuckey-Kramer) revealed that DW ERP amplitudes at Fz during the P50 time range were significantly more positive in the Oddball condition than in the Random (*p* < 0.005) and in the Low confidence (*p* < 0.005) conditions. DW amplitudes during the MMN time range were significantly more negative in the Oddball condition than in the Random (*p* < 0.005) condition.

## Discussion

Predictive coding models propose that sensory event-related brain potentials reflect the transmission of precision-weighted PE from lower to higher areas of the sensory hierarchy. According to this view, electrophysiological deviance detection signals like the MMN reflect the greater amount of PE elicited by the deviant (mispredicted) compared to the standard (predicted) stimuli (Friston, 2005; Garrido et al., 2009). Moreover, the gain of the PE is adjusted on the basis of an estimation of its precision, whereby more variable (uncertain) contexts lead to lower confidence and down-weighted PE signals compared to more stable (certain) contexts. To test whether the amplitude of sensory evoked responses reflecting PE varies as a function of uncertainty, we recorded ERPs elicited by 1046.5 Hz tones presented with *p* = 0.1 and manipulated the degree of variability of the rest of the sounds of the sequence, which were always drawn from a pool of 10 tones ranging between 440 and 2489.02 Hz. We found that the amplitude of the sensory response evoked by a low probability sound correlates linearly with the variability of the sound sequence in which it is embedded. Specifically, the lower the variability, the more negative the evoked response recorded over frontocentral electrodes between 122 and 202 ms. This gradual increase in negativity in the D tone ERP resulted in an MMN response in the D-S difference waves which decreased linearly with decreasing S tone probability. These results provide strong support for the idea that evoked potentials in the time range of the MMN reflect precision-weighted PE.

Traditionally, the MMN has been considered to be automatic and tied to sensory memory, thus operating on short time scales (< 30 s, Winkler et al., 2002) and reflecting local probability statistics (Fitzgerald and Todd, 2020). However, evidence has accumulated indicating that MMN is influenced by higher-order factors such as prior experience, foreknowledge through instruction (Frost et al., 2018), first impression biases (Todd et al., 2011, 2013) and attention (Auksztulewicz and Friston, 2015). These findings challenge the classic views on the computations underlying the MMN, centered on relatively simple mechanisms of deviance detection and regularity extraction, and push toward a broader conceptualization of the MMN as an index of more sophisticated learning processes in a world of sensory uncertainty in which precision plays a key role (Mathys et al., 2011; Fitzgerald and Todd, 2020).

In order to better understand the processes indexed by the MMN, here we have proposed a paradigm studying the impact of precision on sensory deviance detection focusing on the analysis of the D stimulus responses. We have chosen the term precision to refer to our manipulated variable. However, different terms relating to this idea (precision, confidence, uncertainty, variability, signal-to-noise ratio, predictability, context, second-order predictions, etc.) are used somewhat interchangeably in the literature, each stressing slightly different aspects. In general, they all relate to the hypothesis that, to cope with the many factors that limit the reliability of sensory information about the world, the brain encodes information probabilistically, in the form of probability distributions (“Bayesian coding hypothesis,” Knill and Pouget, 2004). These distributions represent all possible values of any parameter, along with the associated probabilities for each value. Uncertainty typically refers to the width of the belief (or subjective probability) distribution (Ma and Jazayeri, 2014), and its inverse is the precision (Feldman and Friston, 2010). Thus, broader distributions (more variance) correspond to greater uncertainty and lower precision. Precision is also often defined as second-order predictions, or the predictions of context (Koelsch et al., 2019; Auksztulewicz and Friston, 2016), referring to contextual factors that influence predictability. That is, besides making a (first-order) prediction on content, the brain would make a (second-order) prediction, based on context, on how predictable an event is, or in other words, how likely it is that the content prediction will be correct (confidence). Therefore, uncertainty can also be defined as a measure of unpredictability or expected surprise (Feldman and Friston, 2010).

Altogether, the degree of variability (unpredictability) stands out as a crucial factor modulating sensory deviance detection, but variability can take myriad different forms. Indeed, different types of uncertainty have been proposed to drive different modulatory processes (Yu and Dayan, 2005), and, in principle, precision can refer to the belief distribution (the model), the predictions derived from it, the PE, or the stimulation itself. Logically, these are interrelated, as, for example, more variable contexts will lead to more uncertain predictions (Mathys et al., 2011). Nevertheless, both the precision of the prediction and the precision of the PE need to be considered to estimate the net effect of the sensory input on each observation (Kwisthout et al., 2017). In fact, the precision-weighted PE can be viewed as a Student’s t-statistic, where to assess the significance of the difference between two distributions (the prediction and the observation) the difference in means (PE) is divided by its standard error (inverse variance or precision) (Feldman and Friston, 2010).

Hence, the question remains, what exactly does precision refer to? What types of variability or uncertainty specifically influence the magnitude of a deviance detection signal like the MMN? Given the multiple perspectives on how to define confidence, it seems necessary to empirically explore different types of manipulations to understand the significance of precision for the MMN.

The degree of predictability has been manipulated in many different ways in deviance detection studies. First of all, the very definition of the MMN as a deviance detection signal implies that it depends on predictability: only when the stimulation contains some type of statistical regularity that can be violated will there be the possibility to elicit an MMN. In fact, presenting the deviant sounds with the same probability embedded in a sequence of random (unpredictable) sounds (our Random condition) is an established control to isolate the MMN (Schröger and Wolff, 1996; Ruhnau et al., 2012). Indeed, Hsu et al. (2015) showed that relative to predicted stimuli, mispredicted stimuli (deviants violating the established regularity) elicited enhanced negative responses while unpredicted stimuli (presented in the absence of a rule) elicit attenuated responses.

Thus, the question is rather whether, when there is a statistical rule to be violated, the amplitude of the deviance detection signals depends on the degree of predictability. Previous studies have manipulated the strength of the rule by manipulating the number of consecutive repetitions of the standard presented immediately before the deviant (Baldeweg et al., 2004; Haenschel et al., 2005; Costa-Faidella et al., 2011a,b), or more generally the degree of repetitiveness of the sequence (Quiroga-Martinez et al., 2019, 2020) reporting greater MMN amplitudes with increased repetition. However, one concern with these types of studies is whether the effects observed reflect a modulation of the deviance detection process, or whether they reflect refractoriness differences.

The strength of the rule can also be weakened by introducing small variations in the characteristics of the repeating standard stimulus. Winkler et al. (1990) varied sound intensity across standard stimulus exemplars. In different blocks, the “substandards” covered a wider or narrower range of intensity values around a common mean. MMN elicited by intensity deviants decreased as the range of variation in the standard increased. In a similar design, Daikhin and Ahissar (2012) found that jittering the standard frequency reduced responses to frequency deviants, but only when the deviance magnitude was small. Importantly, in both these studies the deviants were defined by being outside the range of variation of the standard, thus adaptation differences could play a role in these effects as well, and the standard was always varied, thus no repetition rule was established.

We have proposed a parametric manipulation based on the stability of the standard stimulus, akin to a manipulation of signal-to-noise ratio, directly manipulating the strength of the rule (the rule being the standard tone and the noise being the rest of the tones). Aiming to investigate the effects of precision on the “true” MMN, or the part of the MMN which is due to predictive processes and not local adaptation or refractoriness mechanisms (i.e., repetition effects), we focused on the analysis of the responses to deviant stimuli with identical probability across the different standard stability conditions (a similar strategy to using a random control, Schröger and Wolff, 1996; Ruhnau et al., 2012). The results show a clear gradation of the D response with a time-course and scalp distribution compatible with the MMN. However, the MMN is typically extracted calculating the D-S difference wave, canceling out sensory responses and isolating the deviance detection signal. Therefore, the response elicited by the D stimulus cannot directly be considered an MMN. Both modulations of the D stimulus and the S stimulus responses affect the canonical MMN signal. In classic paradigms, ideally, the S and D responses are extracted from different conditions in a block design, so that they are elicited by the same physical stimulus under the two different roles. In our paradigm, the S and D tones are different physical stimuli. However, the mode of presentation of the D stimulus in our Random condition is identical to how the control S stimulus is presented in the well-established “many standards” or random control condition, used in previous studies to isolate the “true” MMN (Schröger and Wolff, 1996; Ruhnau et al., 2012). Therefore, the difference between the D ERP in the Oddball and the Random conditions is indeed the MMN response, and we can observe a clear gradual modulation of this response across the levels of our parametric precision manipulation.

Nevertheless, to provide a complete picture and for ease of comparison to the previous literature, we also analyzed the S stimulus responses, and found no significant correlation between the S tone ERP and the parametric precision manipulation. While this finding suggests that precision affects only the D and not the S responses, it should be interpreted with caution, as precision effects on the S responses might have been compensated with refractoriness effects, given the manipulation of the S stimulus probability across precision conditions. Thus, albeit precision can of course affect both S and D responses, and both effects would impact the MMN signal, practical issues regarding the design of the paradigm make it quite difficult to study both these aspects at the same time. Here we have focused on the investigation of the effects of precision on the deviance detection signal *per se*, which is elicited by the D, not the S stimulus, and contributes directly to the canonical MMN response.

In any case, again to facilitate comparison to previous studies, we also report the classic D-S difference waves, where the MMN can be clearly identified in the Oddball and the High confidence conditions, as expected. The modulations observed on the MMN response isolated in this way show the same pattern as the modulation observed on the D tone ERP in the MMN time window, with an additional earlier significant modulation affecting the P50 response. Again, given that the difference waves reflect both modulation of the S tone and the D tone ERPs and that the S tone ERP is also affected by refractoriness differences, this result should be interpreted with caution, and we prefer to refrain from making any firm conclusions based on the difference wave ERPs.

Thus, we have based our conclusions on the analysis of the D tone ERPs which had a fixed probability throughout the experiment. Despite their fixed probability, it could be argued that co-adaptation from nearby frequency channels could modulate deviant responses differentially across conditions (Jääskeläinen et al., 2004; May and Tiitinen, 2010; for a review of animal and human studies on stimulus-specific adaptation, see Escera and Malmierca, 2014). However, our results are inconsistent with this hypothesis, as co-adaptation in nearby frequency channels should render the responses to the deviant in the oddball condition smaller than in the rest of experimental conditions. Instead, previous studies have shown that neuronal responses scale with the spectral distribution of auditory stimulation, a finding showing a dynamic variation in stimulus-specific adaptation, interpreted as adaptation to stimulus statistics (Herrmann et al., 2013, 2014). Indeed, several findings indicate that alphabet size (Winkler et al., 1992; Barascud et al., 2016; Auksztulewicz et al., 2017; Quiroga-Martinez et al., 2019, 2020) or the width of the distribution (Garrido et al., 2013; Larsen et al., 2020) of the stimulation sequence are reflected on neural signals, supporting the idea that variability in the stimulation (inverse precision) plays a role in the modulation of deviance detection. In our study, decreasing the confidence (from oddball to random) increases the spectral variability of the stimulation (i.e., tones of different frequencies become more probable), without broadening the spectral range of the sequence. As the low probability stimulus (D) falls at the center of the mean log spectral distribution of the stimulation (1046.5 Hz), the better the model representing the spectral distribution, the more reduced neural responses would be expected (Daikhin and Ahissar, 2012; Garrido et al., 2013) as the D tone becomes a prototype exemplar of the rule. Thus, encoding the distribution of stimulation features, such as tone frequency, could stand as a possible mechanism underlying precision-weighting of PE in variable contexts.

An interesting question is whether variability in one feature affects only deviance detection processes with respect to that feature, or whether reduced model confidence down-weights PE signals arising from violations of any of the stimulation parameters. Here, we have directly manipulated the predictability of the stimulus feature in which the deviant differs from the standard. However, confidence can also be manipulated varying the number of features that are predictable. Some findings suggest that variability in one feature does not affect deviance detection with regards to other features (Quiroga-Martinez et al., 2019, 2020). However, there is also evidence that manipulating the variability in one feature affects the detection of deviations in a second (stable) feature (Winkler et al., 1990). Notably, introducing temporal uncertainty (variability) reduces repetition suppression (Costa-Faidella et al., 2011a) and impairs the ability to detect new rules (Sohoglu and Chait, 2016). Thus, future studies using our paradigm could explore how the spectral variability manipulation across confidence levels affects responses to deviations in other features (e.g., duration or intensity deviants).

The strategies discussed so far modulated precision manipulating always low-level features of the stimulation; that is, physical differences between standards and deviants. However, predictability can also be increased by imposing additional higher-level rules or constraints. When participants are informed about the rules, the MMN is modulated (Frost et al., 2018; but see Koelsch et al., 2019 for an opposing argument). Moreover, stimuli that violate a local rule elicit smaller PE signals if they at the same time conform to a global rule (Sussman et al., 1998; Wacongne et al., 2011). It should be noted that manipulating predictability by imposing a higher order rule, is not the same as directly making the single existing rule more or less noisy. Nevertheless, these studies show that information from different levels of the representation hierarchy is integrated and top-down information from higher levels seems to be able to readjust precision at lower levels. Similarly, recent studies have shown that the MMN is affected by the rule stability estimated over time scales that must necessarily involve higher-order structures. In these studies, volatility is manipulated having the standard and deviant change roles more or less rapidly throughout the stimulation sequence, showing that MMN is larger during more stable stimulation stretches (Todd et al., 2011, 2013; Dzafic et al., 2020).

All in all, studies manipulating predictability in one way or another have shown that deviance detection signals are higher in less variable (more predicable) conditions. However, in general, the studies discussed above made comparisons between certain vs. uncertain conditions, but did not show a gradation of different levels of uncertainty. Thus, it is interesting to understand whether deviance detection is a process that varies parametrically with precision whenever precision is manipulated through the degree of regularity. Alternatively, there could be an “all-or-none” turning point when a given predictive model of the stimulation is accepted as valid, and only from that point on is the system actually using it to make predictions. For example, in a study investigating the effects of deviance magnitude, Horváth et al. (2008) gradually manipulated the frequency distance between deviant and standard and argued that the “true” MMN (when adaptation is controlled) is categorical, an all-or-none process. We performed a gradual manipulation of the rule strength across 4 levels of uncertainty, and found that responses to the deviant scale with rule precision, pointing to a continuous process. Nevertheless, at a descriptive level, we also observed a possible qualitative change between the oddball and the high precision conditions. Topographically, the process that varied parametrically with precision was centrally distributed. However, careful observation of the topographies of the oddball condition suggests the presence of an overimposed frontocentral negativity in this condition only, that is already not observable in the high confidence condition. This change in topography could reflect the activation of frontal generators (Deouell, 2007), suggesting that highly precise PEs may reach higher hierarchical levels before they can be silenced. The presence or absence of filler tones might also represent an important qualitative change in the stimulation leading to different strategies in the deviance detection process. Nevertheless, on the classic D-S difference waves, a clear MMN response can be observed both for the Oddball and the High confidence conditions, while no MMN is elicited in the Random condition, as expected from previous studies, and the signal elicited by the Low confidence condition lies somewhere in between. This indeed seems to suggest that the signal reflects a continuous rather than an all or none underlying process, however, additional research is needed to clarify this point. Specifically, it could be interesting to add more confidence steps to the design to further evaluate the gradation of the responses, and to extend the electrode montage to be able to perform a reliable source analysis, or use a technique with a higher spatial resolution, that would allow dissociating multiple hypothetical contributing sources.

In conclusion, in our paradigm, we have tapped into precision by manipulating pitch predictability gradually, going from random frequencies within a limited range, to a strong (low-level) repetition rule. However, contrary to other studies that have manipulated repetitiveness, we focus on the response to D sounds of equal probability, thereby avoiding adaptation confounds. In our study, decreasing repetitiveness of the S rule means increasing spectral variability, similarly to alphabet size or distribution width manipulations, but critically our D stimulus falls on the center of the distribution and the range of values was equal across conditions, manipulating only the repetitiveness of the S within this range. We show that gradually lowering the precision of the pitch rule, gradually weakens responses to pitch deviants. The results support the view that sensory responses to the D sound are a manifestation of precision-weighted PE, in the context of a Bayesian inference process. However, as we have reviewed, there are various ways to define precision and manipulate it at multiple levels. Further research is needed to clarify whether all these effects reflect the same underlying process or not.

With this paradigm, we hope to demonstrate a viable, gradual manipulation of precision in the investigation of prediction and prediction errors in the auditory modality, which addresses the “true” MMN controlling for adaptation. Experimental manipulations tapping onto precision can be powerful tools to explore predictive processing and learning and their dysfunctions, and can be used to test the hypothesis of aberrant precision-weighting in schizophrenia and autism (Adams et al., 2013; Lawson et al., 2014, 2017; Haarsma et al., 2020). We believe our paradigm can shed some light on the concept of precision and the precision-weighting of prediction error signals in the Bayesian inference process, contributing to continuously advance the understanding of the MMN-generating process toward a broader conceptualization of the MMN as a signal of sophisticated learning processes in a world of sensory uncertainty.

## Data Availability Statement

The raw data supporting the conclusions of this article will be made available by the authors, without undue reservation.

## Ethics Statement

The studies involving human participants were reviewed and approved by the Clinical Research Commission of the Hospital Universitari Institut Pere Mata, Drug Research Ethics Committee of the Institut d’Investigació Sanitària Pere Virgili and Bioethics Committee of the University of Barcelona. The patients/participants provided their written informed consent to participate in this study.

## Author Contributions

ISM, JC-F, and CE conceptualized and designed the paradigm. JC-F programmed the task and analyzed the data. ZL acquired the data. JC-F and ISM wrote the first version of the manuscript. CE, ZL, and EV revised the manuscript. CE and EV supervised the work. All authors contributed to the article and approved the submitted version.

## Conflict of Interest

The authors declare that the research was conducted in the absence of any commercial or financial relationships that could be construed as a potential conflict of interest.

## Publisher’s Note

All claims expressed in this article are solely those of the authors and do not necessarily represent those of their affiliated organizations, or those of the publisher, the editors and the reviewers. Any product that may be evaluated in this article, or claim that may be made by its manufacturer, is not guaranteed or endorsed by the publisher.
